# Rush Medical College Clinics

**Published:** 1873-07

**Authors:** 


***Rush Medical College Clinics.***

A course of Clinical Lectures upon the Diseases of the Brain and Nervous System, will be commenced at Rush Medical College, corner of 18th and Arnold streets, on Saturday, August 2nd, at 3.30 P. M., and will be continued on every succeeding Saturday throughout the year, by Dr. Walter Hay, Adjunct Professor of Theory and Practice of Medicine and Lecturer on Diseases of the Brain and Nervous System in Rush Medical College.

